# Pharmacogenetic Variation and Its Clinical Relevance in a Latin American Rural Population

**DOI:** 10.3390/ijms231911758

**Published:** 2022-10-04

**Authors:** Jordi Olloquequi, Patricia Castro-Santos, Roberto Díaz-Peña

**Affiliations:** 1 Departament de Bioquímica i Fisiologia, Facultat de Farmàcia i Ciències de l’Alimentació, Universitat de Barcelona, 08028 Barcelona, Spain; 2 Laboratorio de Patología Celular y Molecular, Instituto de Ciencias Biomédicas, Facultad de Ciencias de la Salud, Universidad Autónoma de Chile, Talca 340000, Chile; 3 Inmunología, Centro de Investigaciones Biomédicas (CINBIO), Universidad de Vigo, 36310 Vigo, Spain; 4 Fundación Pública Galega de Medicina Xenómica, SERGAS, Grupo de Medicina Xenomica-USC, Instituto de Investigación Sanitaria de Santiago (IDIS), 15706 Santiago de Compostela, Spain

**Keywords:** pharmacogenetics, Latin-American, ancestry, personalized medicine, Chile, single nucleotide polymorphism

## Abstract

Latin-American populations have been largely underrepresented in genomic studies of drug response and disease susceptibility. In this paper, we present a genome-wide Chilean dataset from Talca based on the Illumina Global Screening Array. This let us to compare the frequency of gene variants involved in response to drugs among our population and others, taking data from the 1000 Genomes Project. We found four single-nucleotide polymorphisms with low prevalence in Chileans when compared with African, Amerindian, East and South Asian, and European populations: rs2819742 (*RYR2*), rs2631367 (*SLC22A5*), rs1063320 (*HLA-G*), and rs1042522 (*TP53*). Moreover, two markers showed significant differences between lower and higher proportion of Mapuche ancestry groups: rs1719247 (located in an intergenic region in chromosome 15; *p*-value = 6.17 × 10^−5^, Bonferroni corrected *p*-value = 0.02) and rs738409 (A nonsynonymous gene variant in the *PNPLA3* gene; *p*-value = 9.02 × 10^−5^, Bonferroni corrected *p*-value = 0.04). All of these polymorphisms have been shown to be associated with diverse pathologies, such as asthma, cancer, or chronic hepatitis B, or to be involved in a different response to drugs, such as metformin, HMG-CoA reductase inhibitors, or simvastatin. The present work provides a pharmacogenetic landscape of an understudied Latin American rural population and supports the notion that pharmacogenetic studies in admixed populations should consider ancestry for a higher accuracy of the results. Our study stresses the relevance of the pharmacogenomic research to provide guidance for a better choice of the best treatment for each individual in a population with admixed ancestry.

## 1. Introduction

Pharmacogenetics is the study of variations in genes encoding drug-metabolizing enzymes, drug transporters and drug targets, and their translation to the responses of individuals to drug therapy [1]. The possibility of conducting gene-based testing allows to offer the appropriate medication to different individuals at the right dose, minimizing toxicity and maximizing the efficacy, which in turn results in personalized medicine. This could be achieved studying the polymorphisms of the relevant pharmacogenes and designing personalized profiles that would allow to stratify individuals to different treatment options. In this regard, Pharmacogenomics Knowledge Base (PharmGKB) is a resource that provides information about the impact of variants in pharmacodynamics and pharmacokinetics genes in drug response [2]. Clinical annotations are listed in PharmGKB based on the curated literature, assigning them a level of evidence representing the strength of support for that association. Since the implementation of an individualized treatment depends on the processing of the information obtained at a group level population, the genetic diversity of pharmacodynamics and pharmacokinetics genes between different populations may have biomedical implications [3].

The vast majority of the genetic variants associated with drug response and disease susceptibility have been identified in European populations. In fact, participants in genome-wide association studies (GWAS) are normally ~80% European descent, whereas the representation of Latin American (LA) ancestry is <1% [4]. Given that Latin America and the Caribbean population represents up to ~8.5% of the total world population (https://www.worldometers.info/, accessed on 1 August 2022), the limited availability of genetic studies in Latin Americans is an obstacle to the progress and understanding of pharmacogenetics. Moreover, Hispanic or LA individuals are considered homogenous in most of the GWAS where they are included, but their genomes present different levels of admixture according to the major ancestry population component [5]. Thus, geographically restricted genetic variation may have biomedical implications, that may, in turn, have an impact for the implementation of the personalized medicine in all populations. Differences in the allele frequencies distribution of pharmacogenetic variants, especially between Europeans, Asians, and Africans, entail the risk of extrapolating outcomes from one ethnic group experience to another [6]. When the health authorities approve a new drug in a given nation, many other governments from different geographic regions must address the efficacy and safety variations for their own citizens [7]. However, the relationship between pharmacogenetic variants and drug response, particularly in under-represented populations, is generally bypassed and ignored, although it constitutes a key factor for the development of personalized medicine. For that reason, studies unveiling pharmacogenetic profiles in under-represented populations are still required. In the present study, we determined the allele frequencies distribution of pharmacogenetic variants with significance to precision medicine in a rural Chilean population. Our aim is to provide new data on the pharmacogenomics of an under-studied LA population, in order to contribute to the theoretical basis for future drugs guidance in Chileans and other admixed populations.

## 2. Results

### 2.1. Analysis of Population Structure

**Figure 1:**
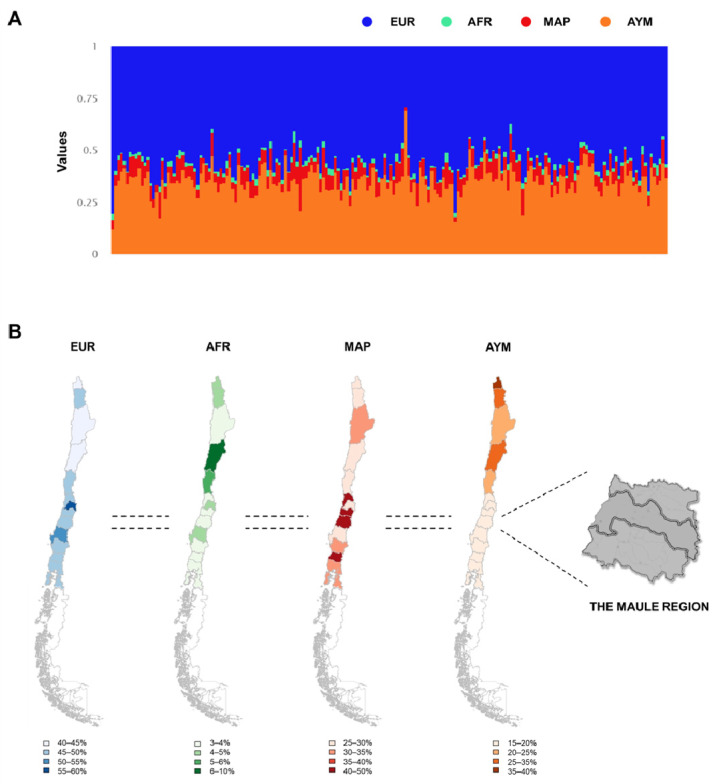
Analysis of Population Structure. (**A**) Estimation of the ancestry proportions of the 190 individuals in this study; (**B**) Maps with average regional African, European, Aymara and Mapuche proportions in Chile.

Estimation of individual ancestries revealed a homogenous Maulean population, with an outstanding influence of the European and Mapuche ancestries (Figure 1A). The average European proportion was 55.94%, whereas the average Mapuche percentage was 35.36%. The average Aymara and African proportions were 6.94% and 1.75%, respectively. We also determined the ancestry proportions at every geographic zone (Figure 1B), illustrating the non-uniformity of the genetic structure of Chileans along the country. El Maule Region have the larger proportion of European ancestries (and smaller proportion of Native-American ancestry). These data indicate that the subjects of this study constitute a representative sample of El Maule Region.

### 2.2. Drug Metabolizing Enzymes and Transporters Variants

A total of 455,564 single-nucleotide polymorphisms (SNPs) were considered for the present study after the following strict quality criteria filtering. We identified 401 clinically relevant drug metabolizing enzymes and transporters (DMET) common variants listed in the PharmGKB database (https://www.pharmgkb.org/, accessed on 1 July 2022) (Supplementary Table S1). Using data from the 1000 Genomes Project [8], we found four SNPs with low prevalence in the Chilean population from Talca when compared with all other world populations (Table 1). Specifically, SNPs were rs2819742, located in an intron of *RYR2* gene (ryanodine receptor 2), rs2631367, located in the promoter region of the *SLC22A5* gene (solute carrier family 22 member 5), rs1063320, located in the 3′-UTR of *HLA-G* gene (major histocompatibility complex, class I, G; one of the non-classical class I HLA molecules) and rs1042522, located in the *TP53* gene (tumor protein p53), in which the G allele encodes an arginine at position 72 of the protein, where there is normally a proline. In addition, the rs2071888-C allele also showed a low prevalence in the Chilean population from Talca compared to Amerindian, European, African and South Asian populations, although not in East Asians. The SNP rs2071888 is a non-synonymous polymorphism in exon 4 of the *TAPBP* gene (TAP binding protein, tapasin), causing an arginine to threonine change (Thr260Arg). On the other hand, rs12943590, located upstream of *SLC47A2* gene (solute carrier family 47 member 2) was found with high prevalence in the Chilean population from Talca when compared with Amerindians, Europeans, and Africans, but not with East and South Asians.

We also investigated the influence of the proportion of Mapuche ancestry (PMA) on the distribution of the pharmacogenetic variants in the Chilean population from Talca (Table 2). We categorized all the individuals included in the study in low PMA or high PMA, based on the median value of the Mapuche percentage (<35% vs. ≥35%). Two markers showed significant differences between lower and higher PMA after Bonferroni correction (P_B_ < 0.05): rs1719247 (located in an intergenic region in chromosome 15; *p* value = 6.17 × 10^−5^, P_B_ = 0.02) and rs738409 (encoding the isoleucine to methionine variant at protein position 148 in patatin-like phospholipase domain-containing protein 3 gene, *PNPLA3*; *p* value = 9.02 × 10^−5^, P_B_ = 0.04). Seven additional markers in seven genes showed suggestive associations (*p* value < 0.05) (Table 2). The associations of the remaining SNPs included in the study were not significant.

### 2.3. Human Leukocyte Antigens

**Figure 2:**
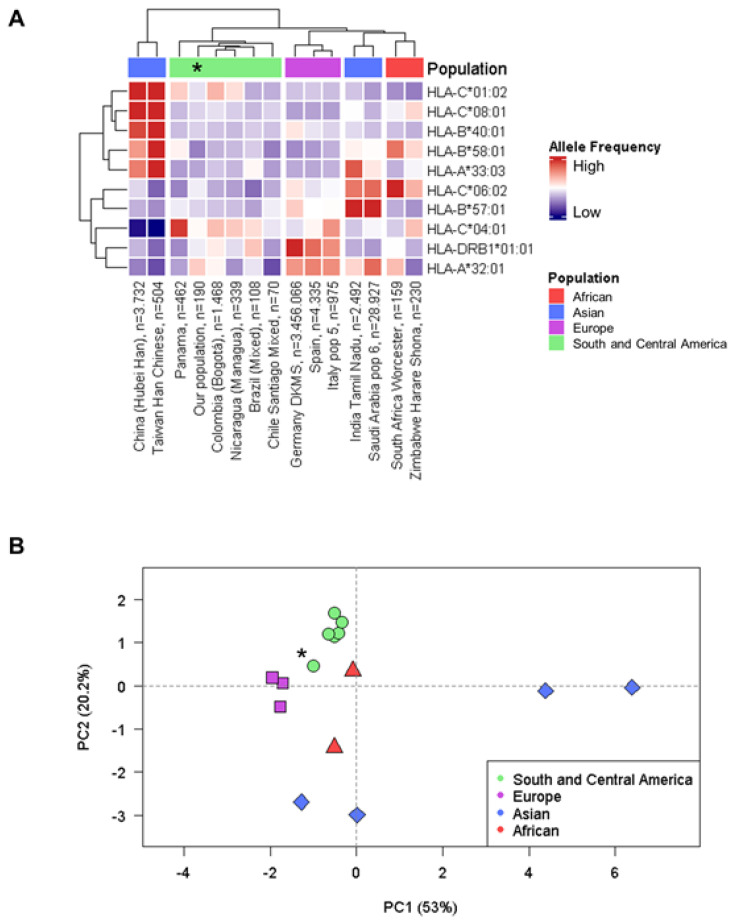
Comparison of HLA allele frequencies in different world populations, including the Chilean population from Talca. (**A**) Hierarchical population clustering; (**B**) Principal component analysis. * The studied population.

We re-examined the distribution of HLA class I and II alleles that were previously described by our group [9]. We evaluated >7000 HLA SNP markers to input HLA alleles, analyzing the frequency of the alleles that have been associated with adverse drug reactions [10], and/or listed in the PharmGKB database (https://www.pharmgkb.org/, accessed on 1 July 2022). Sixty-nine HLA alleles are listed in the Supplementary Table S2, together with the drug or molecule involved, the phenotype (toxicity/efficacy), and the allele frequency in the Chilean population from Talca. Out of the 69 alleles and 45 drugs, 10 HLA alleles and seven drugs showed a high level of evidence, based on PharmGKB database (Table 3). We next compared the HLA allele frequencies of these alleles in the studied population with those from other populations, using the available data in The Allele Frequency Net Database (AFND, www.allelefrequencies.net). Hierarchical clustering and principal component analysis (PCA) are showed in the Figure 2. The Chilean population from Talca clustered separately (Figure 2A), together with other populations from South and Central America. In fact, all groups (African, Asian, European, and South and Central America) grouped differently, taking into account South-East and South-West countries (China/Taiwan, and India/Saudi Arabia). HLA alleles, in general, were quite similar to other populations from Central and South America. We also completed a PCA to explore the relative genetic distance, showing that the Chilean population from Talca was genetically close to South and Central America populations, but also to the European populations (Figure 2B).

## 3. Discussion

Genomic variability among populations can offer new opportunities to detect genetic variants associated with drug response and disease susceptibility. Unfortunately, LA populations have been largely underrepresented in these studies. In this context, the present work provides a genome-wide Chilean dataset from Talca using the Illumina Global Screening Array, allowing to increase our knowledge of the genetic patterns in a rural LA population.

The admixture patterns of the Chileans is determined by the interaction of ancient populations and more recent demographic movements [11]. The first Spaniards arrived in the mid-sixteenth century, and a continuous Caucasian–Native-American miscegenation has taken place since then. The incorporation of a minor African component during the seventeenth century and new migrations coming mainly from Europe over the last two centuries configure the admixture pattern of modern Chileans. The result is a correlation between genetic diversity and geography, in which European ancestry proportions are highest in central regions and African ancestry decreases from North to South [12]. Hence, there is a not uniformly distributed genetic structure along Chile, and this geography-dependent spreading of the alleles requires the implementation of in-depth genetic studies that would allow to truly advance in global medical genomics [13].

Our results corroborate the fact that most European ancestry in Chile can be found in the central region. Talca is the capital of El Maule Region, located within what is considered Central-South Chile. During the first encounters with Spaniards in the XVI century, the region was inhabited by indigenous people [14]. Even during the colonial period, the region increased its diversity due to the presence of European and African populations, and the variability of groups resulting from the genetic exchange. That being the case, it would be logical to find variations in allele frequencies of some SNPs among Chileans and other populations, and some of these SNPs may be involved in drug mechanisms and behaviors. Following this line of thought, our study examined the distribution of 401 pharmacogenomic variants from PharmGKB database in the Chilean population from Talca and compared it with data from the 1000 Genomes Project for five major populations (African, Amerindian, East and South Asian, and European). We identified a number of interesting pharmacogenetic gene variants with low allele frequencies when compared with the other populations, such as rs2819742 (*RYR2*), rs2631367 (*SLC22A5*), rs1063320 (*HLA-G*) and rs1042522 (*TP53*). In addition, rs2071888 (*TAPBP*) showed a different genotype distribution between the Chilean population from Talca and African, Amerindian, European and South Asian populations, whereas the minor allele frequency of rs12943590 (*SLC47A2*) showed the greatest fluctuation between the Chilean population from Talca and African, Amerindian, and European populations.

*RYR2* encodes a ryanodine receptor found in cardiac muscle sarcoplasmic reticulum. This protein is one of the components of calcium channels, that mediates the release of Ca^2+^ from the sarcoplasmic reticulum into the cytoplasm, thus playing a major role in triggering cardiac muscle contraction [15]. There are mutations in *RYR2* gene that have been associated with stress-induced polymorphic ventricular tachycardia and arrhythmogenic right ventricular dysplasia [16,17]. Marciante et al. conducted a study to examine genetic markers for cerivastatin-associated rhabdomyolysis [18], reporting that genotype AA of SNP rs2819742 was associated with decreased risk of rhabdomyolysis when treated with cerivastatin as compared to genotype GG. In a subsequent study, rs2819742-A allele was associated with the development of statin-associated myalgia/myopathy, atorvastatin and simvastatin, in the Czech population [19]. Regarding the organic cation transporter novel type 2 (*OCTN2*), or SLC22A5, it acts as a sodium-dependent transport protein for carnitine in different organs, such as the liver, kidney, and intestine, to eliminate endogenous small organic cations as well as different drugs [20,21]. Mutations in the *SLC22A5* gene are the cause of primary systemic carnitine deficiency [22], and different polymorphisms in the *SLC22A5* gene have been involved in susceptibility to autoimmune diseases, specifically type 1 diabetes and Crohn’s disease [23,24,25]. Moreover, intestinal epithelial OCTN2 expression have been found to be increased in inflamed regions [26], highlighting their role in the intestinal homeostasis. Thus, Angelini et al. showed that rs2631367-G allele was associated with increased response to imatinib in people with gastrointestinal stromal tumors [27], although a later study did not replicate this observation [28]. Further investigations are required in this regard. In turn, HLA-G is a nonclassical HLA class I molecule that exerts a regulatory function. Although it shows a restricted tissue expression pattern, with a relevant role in the maintenance of fetal–maternal immune tolerance [29], HLA-G expression has been associated with different diseases, particularly autoimmunity and cancer [30]. In addition, SNP rs1063320 genotype have been associated with frequency of asthma exacerbations in statin users [31]. Interestingly, the authors showed that rs1063320-G allele was associated with a decreased likelihood of asthma exacerbations in patients exposed to HMG Co-A reductase inhibitors when compared to patients carrying the rs1063320-C allele. This supports the idea that rs1063320 can modify the effect of statin therapy in asthma. On another front, TP53 is a key tumor suppressor gene that plays a crucial regulatory role cell growth, DNA repair and apoptosis [32]. Consequently, the SNP rs1042522 has been reported to be associated with predisposition and clinical outcome in different types of cancer [33], but also with drug resistance in gastric, ovarian and breast cancers [34,35,36,37,38,39]. As for tapasin (*TAPBP*), it constitutes an integral component of the peptide-loading complex that allows HLA-I to present antigens on the cell surface [40]. While a reduction in TAPBP expression has been associated to tumor progression in colorectal cancer and chronic hepatitis B [41,42], the rs2071888 SNP has been associated with wild type gastrointestinal tumors and gallbladder cancer [43,44]. Furthermore, asthmatic patients carrying the CG and GG genotypes may have a higher decline in forced expiratory volume in 1 s after aspirin provocation when compared to those patients carrying the GG genotype [45]. Finally, the *SLC47A2* gene is involved in the excretion of toxic electrolytes in urine and bile [46], and its rs12943590 SNP has been largely found to be involved in the pharmacodynamics of metformin and pharmacogenetics of type II diabetes mellitus [47,48].

We also found that Amerindian ancestry is relevant when we analyze the allele frequencies distribution of pharmacogenetic variants in an admixed population like the present one, because some alleles may have specificity of ancestry. Hence, a significant association was detected in the genotype’s distribution of the SNPs rs1719247 and rs738409 between individuals with lower and higher PMA. Previously, Mangravite et al. showed that the rs1719247-T allele was associated with a decreased likelihood of muscular diseases when treated with HMG-CoA reductase inhibitors or simvastatin as compared to rs1719247-C allele [49]. Regarding the SNP rs738409, it belongs to the *PNPLA3* gene, which encodes a triacylglycerol lipase that mediates triacylglycerol hydrolysis in adipocytes, and may play a role in the acyl-chain remodeling of triglycerides [50,51]. The rs738409-G allele is an important genetic risk factor for steatosis, cirrhosis, and hepatocellular carcinoma [52]. Consistently, Liu et al. showed that rs738409-G allele was associated with higher alanine transaminase (ALT) levels after induction therapy in children with acute lymphoblastic leukemia [53]. Moreover, CG + GG genotypes were associated with an increased risk of toxic liver disease after treatment with asparaginase, cyclophosphamide, cytarabine, daunorubicin, mercaptopurine, prednisolone, and vincristine. Interestingly, the impact of SNP rs738409 on ALT elevation differed by race. These observations suggest that pharmacogenomic research of this variant may help to provide guidance for individualized drug use for admixed populations.

The proteins encoded by HLAs are involved in the immune defense against invading pathogens, differentiating self-cells and non-self-cells by presenting processed peptides to T cells [54]. A hallmark of the HLA region is that it is considered to be the most polymorphic region in the human genome [55]. Several studies have reported the relation between HLA allelic variants and adverse drug reactions, providing the necessary evidences to promote the clinical implementation of HLA testing for the prevention of these events in susceptible patients [56]. Overall, the HLA alleles frequency in our population is very similar to other populations from Central and South America. Our PCA plot, however, reveals that the Chilean samples from Talca are genetically close to both South and Central America, and European populations. This, combined with the variability in the patterns of distribution in HLA-A and HLA-B allotypes among Native populations from Latin America [57], suggests that the percentage of ancestry should be taken into consideration when laying the foundations for safer and more effective drug administration. Indeed, taking Chile as an example, we may find important differences in this type of analysis, due to the existence of the aforementioned gradient in European and Amerindian percentages of ancestry from North to South.

We must acknowledge that the sample size is relatively low, and a validation study should be performed prospectively by enrolling new individuals meeting the inclusion criteria in other Chilean regions. However, the robustness of our statistical analysis allows us to consign the genetic variability described. Another limitation of our study is the fact that we could not analyze all the pharmacogenetic variants reported in PharmGKB, but only those included in our dataset. Nevertheless, we provide valuable data concerning 401 clinically relevant variables in an under-studied population. There is a lack of information about the relationship between actionable pharmacogenetic variants and response to drugs Latin American populations. However, international efforts to shorten the region’s gap of information are being carried out [58].

## 4. Materials and Methods

### 4.1. Study Population

This study involved 190 unrelated individuals, with no record of any specific illness, recruited at the Hospital Regional de Talca (Talca, Chile) through a volunteer recruitment program. All participants were living in El Maule Region, which is one of the most rural counties in Chile. The protocol of the study was approved by the Ethical Committee of “Servicio de Salud del Maule” (approval code: 063/15), Chile, and all subjects gave written informed consent prior to enrolling in the study. To show the ancestry proportions at every geographic zone from Chile, we collected data from chilegenomico (http://www.chilegenomico.cl/, accessed on 1 March 2022) and the study carried out by Lorenzo-Bermejo et al. [59].

### 4.2. Genotyping and HLA Imputation

A total of 10 milliliters from whole blood was collected into plastic vacutainer tubes containing ethylenediaminetetraacetic acid (EDTA), and DNA was extracted using the GeneJET Genomic DNA purification kit #K0722 (ThermoScientific, Waltham, MA, USA), following the manufacturer’s protocols. Subsequent to DNA quality control, samples were genotyped using the Illumina Global Screening Array [60], which enabled genotyping of 754,159 genomic markers. We assessed the distribution of DMET variants considering the genetic ancestry, which was previously described and calculated [59,61]. After that, we compared the genotype frequencies in the Chilean population from Talca with those of five major populations from the 1000 Genomes Project (https://www.internationalgenome.org/, accessed on 1 March 2022) [8]: African, Amerindian, East and South Asian, and European. Finally, the classic HLA alleles at HLA-A, B, C, DPB1, DQA1, DQB1, and DRB1 were imputed using HLA Genotype Imputation with Attribute Bagging (HIBAG) using the Hispanic reference data set [62]. AFND (www.allelefrequencies.net, accessed on 1 March 2022) was used to extract HLA allele frequencies for other populations.

### 4.3. Statistical Analysis

Quality control and statistical analysis of data from SNP genotyping was carried out using PLINK (v1.9) [63] and R software (https://www.r-project.org/, accessed on 1 March 2022). The following quality criteria were used for the SNP genotyping data: minor allele frequency (MAF) <0.01, Hardy–Weinberg equilibrium (HWE) *p* < 0.001, and/or missingness >0.2. Pophelper r package was used to visualize population structure [64].

## 5. Conclusions

The present work provides a pharmacogenetic landscape of an understudied LA rural population. We report the distribution of allele frequencies of pharmacogenetic variants that can be related to clinical outcomes. We also analyzed the effect of genetic ancestry and admixture in this distribution. Although additional functional studies should be conducted to confirm the relevance of the variants described, more studies like this one are necessary, since they will contribute to provide a theoretical basis for a safer and fairer drug administration with better therapeutic effects.

## Figures and Tables

**Table 1:** Different frequency of pharmacogenetic variants in our Chilean population compared with all other world populations.

CHR	SNP	Associated Gene	A1	AF A1	AF A1 AMR	AF A1 EAS	AF A1 EUR	AF A1 SAS	AF A1 AFR	* Type of Drug Effect	* Drug
1	rs2819742	*RYR2*	G	0.50	0.66	0.94	0.60	0.83	0.98	Toxicity/ ADR	cerivastatin
5	rs2631367	*SLC22A5*	G	0.34	0.67	0.99	0.56	0.89	0.58	Efficacy	imatinib
6	rs1063320	*HLA-G*	G	0.30	0.55	0.61	0.46	0.73	0.62	Efficacy	Hmg-CoA reductase inhibitors
6	rs2071888	*TAPBP*	C	0.40	0.57	0.42	0.52	0.64	0.76	Toxicity/ ADR	aspirin
17	rs12943590	*SLC47A2*	A	0.45	0.32	0.45	0.27	0.40	0.18	Metabolism/ PK	metformin
17	rs1042522	*TP53*	C	0.21	0.68	0.59	0.71	0.51	0.33	Efficacy, Toxicity/ ADR	antineoplastic agents

Abbreviations: A1, allele 1; ADR, adverse drug reactions; AF, allele frequency; AFR, African; AMR, Amerindian; CHR, chromosome; EAS, East Asian; EUR, European; PK, pharmacokinetics; SAS, South Asian; SNP, single nucleotide polymorphism. * Data from PharmGKB database (https://www.pharmgkb.org/, accessed on 1 July 2022).

**Table 2:** Association analysis of single nucleotide polymorphism markers in participants with high and low proportion of Mapuche ancestry.

				Minor Allele Frequency						
CHR	SNP	Associated Gene	A1	High PMA	Low PMA	OR (95%CI)	*p* Value	P_B_	* Type of Drug Effect	* Drug
15	rs1719247	intergenic	C	0.34	0.54	0.43 (0.29–0.65)	6.17 × 10^−5^	0.02	Toxicity/ADR	Hmg-CoA reductase inhibitors, simvastatin
22	rs738409	*PNPLA3*	G	0.32	0.52	0.44 (0.29–0.66)	9.02 × 10^−5^	0.04	Toxicity/ADR	asparaginase, cyclophosphamide, daunorubicin, prednisolone, vincristine
19	rs10420097	*ZNF211*	G	0.08	0.005	16.03 (2.10–122.60)	3.78 × 10^−4^	0.15	Efficacy	methylphenidate
7	rs6977820	*DPP6*	T	0.181	0.32	0.46 (0.28–0.74)	1.33 × 10^−3^	0.53	Toxicity/ADR	antipsychotics
6	rs3130501	*POU5F1*	A	0.32	0.19	1.97 (1.22–3.16)	4.78 × 10^−3^	1	Toxicity/ADR	allopurinol
9	rs2289658	*NTRK2*	G	0.21	0.11	2.16 (1.22–3.82)	7.23 × 10^−3^	1	Dosage	methadone
2	rs2241883	*FABP1*	C	0.36	0.23	1.84 (1.17–2.87)	7.53 × 10^−3^	1	Efficacy	fenofibrate
6	rs628031	*SLC22A1*	A	0.17	0.28	0.53 (0.32–0.86)	0.01	1	Efficacy	metformin
5	rs2546890	*IL12B*	A	0.46	0.34	1.71 (1.13–2.60)	0.01	1	Efficacy	TNF-alpha inhibitors

Abbreviations: A1, minor allele nucleotide; ADR, adverse drug reactions; CHR, chromosome; CI, confidence intervals; OR, odd ratio; PMA, proportion of Mapuche ancestry; SNP, single nucleotide polymorphism; TNF, tumor necrosis factor alpha. * Data from PharmGKB database (https://www.pharmgkb.org/, accessed on 1 July 2022).

**Table 3:** List of Adverse drug reactions-related HLA alleles and drugs.

Drug	HLA Allele	AF Chilean	* Level of Evidence	ADR
Carbamazepine	HLA-A*32:01	2.89	1A	Toxicity
Allopurinol	HLA-A*33:03	0.26	2B	Toxicity
Carbamazepine	HLA-B*40:01	1.58	2A	Toxicity
Abacavir	HLA-B*57:01	1.58	1A	Drug Hypersensitivity
Flucloxacillin			1A	Toxicity
Allopurinol	HLA-B*58:01	0.53	1A	Drug Hypersensitivity, SJS, TEN
Methazolamide	HLA-C*01:02	5.00	2B	Toxicity
Nevirapine	HLA-C*04:01	12.63	2B	Toxicity
Sulfamethoxazole-Trimethoprim	HLA-C*06:02	7.89	2B	Toxicity
Sulfamethoxazole-Trimethoprim	HLA-C*08:01	1.05	2B	Toxicity
Nevirapine	HLA-DRB1*01:01	3.16	2B	Toxicity

Abbreviations: ADR, adverse drug reactions; HLA, human leukocyte antigen. * The level of evidence was verified from PharmGKB (https://www.pharmgkb.org/, accessed on 1 July 2022).

## Data Availability

The data presented in this study are available on request from the corresponding author.

## Supplementary Materials

The following supporting information can be downloaded at: https://www.mdpi.com/article/10.3390/ijms231911758/s1.
