# Supplementary material for: Pharmacogenetic Variation and Its Clinical Relevance in a Latin American Rural Population
Source: Int J Mol Sci. 2022 Oct 4;23(19):11758. doi: 10.3390/ijms231911758 (PMC9570141; doi:10.3390/ijms231911758)
Supplement: Supplementary file 1 [file ijms-23-11758-s001.zip › ijms-1913748-supplementary.pdf]

Supplementary Material

Table S1. List of drug metabolizing enzymes and transporters common variants analyzed in the Chilean population.

CHR	SNP	Associated Gene	A1	A2	AF_A1	AF_A2	Type	Level of evidence	Chemicals	phenotypes
1	rs16851030	*ADORA1*	T	C	0.08021	0.91979	Toxicity/ADR	3	aspirin	Asthma
1	rs11122576	*AGT*	G	A	0.1579	0.8421	Efficacy	4	amlodipine,chlorthalidone,lisinopril	
1	rs5050	*AGT*	G	T	0.2816	0.7184	Toxicity/ADR	3	aspirin	
1	rs5051	*AGT*	C	T	0.3789	0.6211	Efficacy	3	atenolol	Hypertension
1	rs17602729	*AMPD1*	T	C	0.05263	0.94737	Toxicity/ADR	3	regadenoson	
1	rs2072671	*CDA*	C	A	0.3658	0.6342	Toxicity/ADR	3	cytarabine	Leukemia,Lymphoma
1	rs3215400	*CDA*	I	D	0.4974	0.5026	Toxicity/ADR	3	cytarabine	
1	rs471760	*CDA*	T	C	0.3658	0.6342	Toxicity/ADR	4	gemcitabine	Neutropenia,Pancreatic Neoplasms
1	rs602950	*CDA*	C	T	0.3506	0.6494	Toxicity/ADR	3	capecitabine	Neoplasms
1	rs646776	*CELSR2*	G	A	0.2079	0.7921	Efficacy	3	hmg coa reductase inhibitors	
1	rs800292	*CFH*	T	C	0.3605	0.6395	Efficacy	3	bevacizumab	
1	rs1801133	CLCN6, *MTHFR*	T	C	0.4632	0.5368	Toxicity/ADR	3	antipsychotics	Schizophrenia
1	rs2808630	*CRP*	C	T	0.2698	0.7302	Efficacy	3	rosuvastatin	Coronary Disease
1	rs983332	*CSRP3*	A	C	0.1474	0.8526	Efficacy	3	Tumor necrosis factor alpha (TNF-alpha) inhibitors	Arthritis, Rheumatoid
1	rs4646487	*CYP4B1*	T	C	0.1105	0.8895	Toxicity/ADR	3	docetaxel,thalidomide	Prostatic Neoplasms
1	rs1801158	*DPYD*	A	G	0.01316	0.98684	Toxicity/ADR	3	capecitabine,fluorouracil	Neoplasms
1	rs1801159	*DPYD*	G	A	0.3053	0.6947	Efficacy,Toxicity/ADR,Metabolism/PK	3	capecitabine,fluorouracil,Pyrimidine analogues,tegafur	Neoplasms

1	rs1801160	*DPYD*	A	G	0.04497	0.95503	Toxicity/ADR,Metabolism/PK	3	capecitabine,fluorouracil,s 1 (combination)	Neoplasms
1	rs1801265	*DPYD*	C	T	0.1858	0.8142	Toxicity/ADR	3	fluorouracil	Neoplasms
1	rs2297595	*DPYD*	C	T	0.06053	0.93947	Toxicity/ADR,Metabolism/PK	3	capecitabine,fluorouracil,Pyrimidine analogues,s 1 (combination)	Neoplasms
1	rs1051740	*EPHX1*	C	T	0.3395	0.6605	Dosage	2B	carbamazepine	Epilepsy
1	rs2234922	*EPHX1*	G	A	0.1289	0.8711	Dosage	2B	carbamazepine	Epilepsy
1	rs324420	*FAAH*	A	C	0.3553	0.6447	Other	3	methamphetamine	Substance-Related Disorders
1	rs11587213	*FCER1G*	G	A	0.1184	0.8816	Toxicity/ADR	3	aspirin	Asthma
1	rs12720462	*FMO1*	A	C	0.2947	0.7053	Other	3	olanzapine	
1	rs1736557	*FMO3*	A	G	0.06053	0.93947	Efficacy	3	rosuvastatin	
1	rs2266780	*FMO3*	G	A	0.07368	0.92632	Other	3	olanzapine	
1	rs2266782	*FMO3*	A	G	0.2447	0.7553	Other	3	itopride	
1	rs2144300	*GALNT2*	C	T	0.4263	0.5737	Other	3	atenolol	Hypertension
1	rs1741981	*HDAC1*	C	T	0.2132	0.7868	Efficacy	3	corticosteroids	Asthma
1	rs1800871	*IL10*	T	C	0.3263	0.6737	Dosage,Efficacy	3	tacrolimus	Kidney Transplantation
1	rs11209026	*IL23R*	A	G	0.04211	0.95789	Toxicity/ADR	3	Tumor necrosis factor alpha (TNF-alpha) inhibitors	Psoriasis
1	rs3219484	*MUTYH*	A	G	0.02368	0.97632	Toxicity/ADR	3	cisplatin,cyclophosphamide	Ovarian Neoplasms
1	rs10494366	*NOS1AP*	G	T	0.4842	0.5158	Toxicity/ADR	3	verapamil	Acquired Long QT Syndrome (aLQTS)
1	rs5065	*NPPA*	G	A	0.06842	0.93158	Efficacy	4	amlodipine,chlorthalidone	Cardiovascular Diseases,Coronary Disease,Stroke
1	rs2768759	*NTRK1*	A	C	0.4289	0.5711	Efficacy	3	aspirin	
1	rs12566888	*PEAR1*	T	G	0.1632	0.8368	Efficacy	4	ticagrelor	
1	rs340874	*PROX1*	G	A	0.3895	0.6105	Toxicity/ADR	3	atenolol	Hypertension
1	rs2819742	*RYR2*	G	A	0.4974	0.5026	Toxicity/ADR	3	cerivastatin	Rhabdomyolysis
1	rs10494227	*ZNF697*	G	A	0.07407	0.92593	Efficacy	3	interferon beta-1a,interferon	Multiple Sclerosis

									beta-1b	
1	rs12118636	-	A	G	0.04211	0.95789	Efficacy	3	carboplatin,cisplatin,gemcitabine	Carcinoma, Non-Small-Cell Lung
1	rs12143842	-	T	C	0.2553	0.7447	Efficacy	4	amlodipine,chlorthalidone,lisinopril	
2	rs55754655	*AOX1*	G	A	0.06316	0.93684	Efficacy	3	azathioprine	Inflammatory Bowel Diseases
2	rs676210	*APOB*	A	G	0.2342	0.7658	Efficacy	3	fenofibrate	Hypertriglyceridemia
2	rs10210302	*ATG16L1*	T	C	0.2921	0.7079	Efficacy	3	adalimumab	Crohn Disease
2	rs13393173	*CERS6*	A	G	0.1658	0.8342	Efficacy	3	Tumor necrosis factor alpha (TNF-alpha) inhibitors	Arthritis, Rheumatoid
2	rs3087243	*CTLA4*	G	A	0.4895	0.5105	Toxicity/ADR	3	Tumor necrosis factor alpha (TNF-alpha) inhibitors	Psoriasis
2	rs2304429	*DNMT3A*	G	A	0.4947	0.5053	Dosage	3	warfarin	heart valve replacement
2	rs2241883	*FABP1*	C	T	0.2974	0.7026	Efficacy	3	fenofibrate	Hypertriglyceridemia
2	rs757978	*FARP2*	T	C	0.09211	0.90789	Efficacy	4	methylphenidate	Attention Deficit Disorder with Hyperactivity
2	rs17583889	*HNMT*	A	C	0.1	0.9	Toxicity/ADR	3	anthracyclines and related substances	Neoplasms
2	rs1804645	*NCOA1*	T	C	0.01053	0.98947	Toxicity/ADR	3	tamoxifen	Breast Neoplasms
2	rs17183814	*SCN2A*	A	G	0.08158	0.91842	Efficacy	3	antiepileptics,carbamazepine,phenobarbital,phenytoin,valproic acid	Epilepsy
2	rs7574865	*STAT4*	T	G	0.3789	0.6211	Efficacy	3	etanercept	Arthritis, Rheumatoid
2	rs6749447	*STK39*	G	T	0.4895	0.5105	Efficacy	3	losartan	
2	rs7582141	*TANC1*	T	G	0.1005	0.8995	Toxicity/ADR	2B	radiotherapy	Prostatic Neoplasms
2	rs1042640	*UGT1A*	G	C	0.2579	0.7421	Toxicity/ADR	3	acetaminophen	Liver Failure, Acute
2	rs10929303	*UGT1A*	T	C	0.2737	0.7263	Toxicity/ADR	3	acetaminophen	Liver Failure, Acute
2	rs8330	*UGT1A*	G	C	0.2751	0.7249	Other	4	acetaminophen	
2	rs10929302	*UGT1A1*	A	G	0.3222	0.6778		3	irinotecan	
2	rs4124874	*UGT1A1*	C	A	0.4474	0.5526	Toxicity/ADR	3	irinotecan	Lymphoma,Neoplasms

2	rs4148323	*UGT1A1*	A	G	0.01579	0.98421	Other	2A	SN-38	Neoplasms
2	rs4148324	*UGT1A1, UGT1A10, UGT1A3, UGT1A4, UGT1A5, UGT1A6, UGT1A7, UGT1A8, UGT1A9*	G	T	0.3447	0.6553	Metabolism/PK	3	methotrexate	Osteosarcoma
2	rs2070959	*UGT1A10, UGT1A6, UGT1A7, UGT1A8, UGT1A9*	G	A	0.2026	0.7974	Dosage	3	valproic acid	Epilepsy
2	rs6755571	*UGT1A4*	A	C	0.02105	0.97895	Other	3	ABT-751	Neoplasms
2	rs1105879	*UGT1A6*	G	T	0.2737	0.7263	Dosage	3	valproic acid	Epilepsy
2	rs17863783	*UGT1A6*	T	G	0.07105	0.92895	Toxicity/ADR	3	anthracyclines and related substances	Neoplasms
2	rs7586110	*UGT1A7*	G	T	0.2263	0.7737	Toxicity/ADR	3	atazanavir,ritonavir	HIV,Hyperbilirubinemia
2	rs1344706	*ZNF804A*	G	T	0.4026	0.5974	Efficacy	3	antipsychotics	Schizophrenia
2	rs13432159	-	G	T	0.04211	0.95789	Toxicity/ADR	3	sertraline	Depressive Disorder, Major
2	rs2952768	-	C	T	0.4658	0.5342	Dosage	2B	buprenorphine,fentanyl,meperidine,morphine,opioids,pentazocine	
3	rs13064411	*CFAP44*	G	A	0.08684	0.91316	Efficacy	3	hmg coa reductase inhibitors	
3	rs167771	*DRD3*	G	A	0.25	0.75	Toxicity/ADR	3	risperidone	Bipolar Disorder,Schizophrenia
3	rs1470579	*IGF2BP2*	C	A	0.3053	0.6947	Efficacy	3	repaglinide	Diabetes Mellitus, Type 2
3	rs4402960	*IGF2BP2*	T	G	0.3053	0.6947	Efficacy	3	repaglinide	Diabetes Mellitus, Type 2
3	rs2535629	*ITIH3*	T	C	0.3895	0.6105	Efficacy	3	clozapine	Schizophrenia
3	rs710446	*KNG1*	G	A	0.4342	0.5658	Toxicity/ADR	3	hormonal contraceptives for systemic use	

3	rs6853	*MYD88*	G	A	0.08947	0.91053	Toxicity/ADR	3	fentanyl	
3	rs6785930	*P2RY12*	A	G	0.3368	0.6632	Efficacy,Toxicity/ADR	3	clopidogrel	
3	rs1801282	*PPARG*	G	C	0.1053	0.8947	Toxicity/ADR	3	olanzapine	Schizophrenia
3	rs2742417	*SACM1L*	C	T	0.4816	0.5184	Toxicity/ADR	3	bupropion	Depressive Disorder, Major
3	rs1799852	*TF*	T	C	0.1243	0.8757	Efficacy	4	adalimumab	Crohn Disease
3	rs7624766	-	G	A	0.4734	0.5266	Efficacy	4	methotrexate	Arthritis, Rheumatoid
4	rs12505410	*ABCG2*	G	T	0.4122	0.5878	Efficacy	3	imatinib	Leukemia, Myelogenous, Chronic, BCR-ABL Positive
4	rs2231142	*ABCG2*	A	C	0.09737	0.90263	Efficacy	2A	rosuvastatin	Hypercholesterolemia,Myocardial Infarction
4	rs4148155	*ABCG2*	C	T	0.09788	0.90212	Efficacy	4	allopurinol	
4	rs4961	*ADD1*	T	G	0.1842	0.8158	Efficacy	2B	furosemide,spironolactone	Liver Cirrhosis
4	rs1229984	*ADH1B*	A	G	0.04696	0.95304	Metabolism/PK	3	ethanol	
4	rs729147	*ADH7*	G	A	0.4605	0.5395	Toxicity/ADR	3	anthracyclines and related substances	
4	rs437943	*ARAP2*	G	A	0.4184	0.5816	Efficacy	4	Tumor necrosis factor alpha (TNF-alpha) inhibitors	Arthritis, Rheumatoid
4	rs4444903	*EGF*	A	G	0.4474	0.5526	Efficacy	2B	cetuximab	Colorectal Neoplasms,Rectal Neoplasms
4	rs2289252	*F11*	T	C	0.3921	0.6079	Toxicity/ADR	3	hormonal contraceptives for systemic use	
4	rs1495509	*KCNIP4*	C	T	0.2316	0.7684	Toxicity/ADR	3	Ace Inhibitors, Plain	
4	rs7661530	*KCNIP4*	T	C	0.2632	0.7368	Toxicity/ADR	3	Ace Inhibitors, Plain	
4	rs1364805	*LOC105377356*	T	G	0.2526	0.7474	Efficacy	3	montelukast	Asthma
4	rs1816702	*TLR2*	T	C	0.07895	0.92105	Efficacy	3	Tumor necrosis factor alpha (TNF-alpha) inhibitors	Crohn Disease
4	rs3775291	*TLR3*	A	G	0.3947	0.6053	Efficacy	3	Measles vaccines	
4	rs7662029	*UGT2B7*	A	G	0.3842	0.6158	Efficacy,Toxicity/ADR	3	methadone	Opioid-Related Disorders

4	rs7668258	*UGT2B7*	T	C	0.3842	0.6158	Efficacy,Toxicity/ADR	3	methadone	Opioid-Related Disorders
5	rs4702484	*ADCY2*	T	C	0.3237	0.6763	Efficacy	4	capecitabine	Colorectal Neoplasms
5	rs1650697	*DHFR*	T	C	0.2211	0.7789	Toxicity/ADR	3	pemetrexed	Carcinoma, Non-Small-Cell Lung,Mesothelioma
5	rs4532	*DRD1*	C	T	0.2289	0.7711	Toxicity/ADR	3	dextroamphetamine,methylphenidate	Attention Deficit Disorder with Hyperactivity
5	rs1801020	*F12*	T	C	0.35	0.65	Efficacy	3	Enzymes	Stroke
5	rs351855	*FGFR4*	T	C	0.3474	0.6526	Efficacy	3	cyclophosphamide,fluorouracil, methotrexate	Breast Neoplasms
5	rs12654264	*HMGCR*	T	A	0.3447	0.6553	Other	3	hmg coa reductase inhibitors	Colonic Neoplasms
5	rs17238540	*HMGCR*	G	T	0.02895	0.97105	Efficacy	3	pravastatin	
5	rs17671591	*HMGCR*	T	C	0.2737	0.7263	Efficacy	3	atorvastatin	Hypercholesterolemia
5	rs3846662	*HMGCR*	C	T	0.3921	0.6079	Other	4	simvastatin	Cardiovascular Diseases
5	rs2546890	*IL12B*	A	G	0.4	0.6	Efficacy	3	Tumor necrosis factor alpha (TNF-alpha) inhibitors	Psoriasis
5	rs3213094	*IL12B*	A	G	0.3947	0.6053	Efficacy	3	ustekinumab	Psoriasis
5	rs1295686	*IL13*	A	G	0.377	0.623	Efficacy	3	Hepatitis vaccines	
5	rs924607	*LOC105374608, LOC100996325*	T	C	0.4101	0.5899	Toxicity/ADR	2B	vincristine	Precursor Cell Lymphoblastic Leukemia-Lymphoma
5	rs1801394	*MTRR*	G	A	0.3263	0.6737	Toxicity/ADR	2B	methotrexate	Precursor Cell Lymphoblastic Leukemia-Lymphoma
5	rs2562519	*POLR3G*	G	A	0.2263	0.7737	Toxicity/ADR	3	nevirapine	HIV
5	rs2631367	*SLC22A5*	G	C	0.3378	0.6622	Efficacy	3	imatinib	Gastrointestinal Stromal Tumors
5	rs11960832	*SV2C*	T	C	0.4105	0.5895	Efficacy	3	olanzapine	Schizophrenia
5	rs2075685	*TMEM167A, XRCC4*	T	G	0.3184	0.6816	Efficacy	3	fluorouracil,Platinum compounds,radiotherapy	Stomach Neoplasms
5	rs12054895	-	T	G	0.3947	0.6053	Efficacy	3	citalopram,escitalopram	Depressive Disorder, Major
6	rs2781659	*ARG1*	G	A	0.3316	0.6684	Efficacy	3	selective beta-2-adrenoreceptor	Asthma

									agonists	
6	rs209474	*BRD2*	G	A	0.266	0.734	Efficacy	3	lithium	Bipolar Disorder
6	rs1265112	*CCHCR1*	G	A	0.2698	0.7302	Toxicity/ADR	3	nevirapine	HIV
6	rs130072	*CCHCR1*	A	G	0.1026	0.8974	Toxicity/ADR	3	nevirapine	HIV
6	rs746647	*CCHCR1*	C	T	0.2684	0.7316	Toxicity/ADR	2B	nevirapine	HIV
6	rs6908425	*CDKAL1*	T	C	0.3	0.7	Efficacy	3	Tumor necrosis factor alpha (TNF-alpha) inhibitors	Psoriasis
6	rs7754840	*CDKAL1*	C	G	0.2868	0.7132	Efficacy	3	Dipeptidyl peptidase 4 (DPP-4) inhibitors	Diabetes Mellitus
6	rs7756992	*CDKAL1*	G	A	0.3211	0.6789	Efficacy	3	Dipeptidyl peptidase 4 (DPP-4) inhibitors	Diabetes Mellitus
6	rs806368	*CNR1*	C	T	0.3395	0.6605	Other	3	cocaine	Cocaine-Related Disorders
6	rs2734583	*DDX39B, ATP6V1G2-DDX39B, SNORD117*	C	T	0.04762	0.95238	Toxicity/ADR	3	allopurinol	drug reaction with eosinophilia and systemic symptoms,Stevens-Johnson Syndrome
6	rs5370	*EDN1*	T	G	0.2079	0.7921	Toxicity/ADR	3	muraglitazar	Diabetes Mellitus,Edema,Hyperlipidemias
6	rs5985	*F13A1*	T	G	0.2763	0.7237	Efficacy	3	aspirin	
6	rs451774	*GPX5*	G	A	0.3658	0.6342	Efficacy	3	carboplatin,cisplatin,gemcitabine	Carcinoma, Non-Small-Cell Lung
6	rs2523864	*HCG22*	A	G	0.3789	0.6211	Toxicity/ADR	3	triamcinolone	Retinal Diseases
6	rs12191877	*HLA-C*	T	C	0.1176	0.8824	Efficacy	3	Tumor necrosis factor alpha (TNF-alpha) inhibitors	Psoriasis
6	rs9461684	*HLA-C*	T	C	0.1263	0.8737	Toxicity/ADR	3	nevirapine	HIV,HIV Infections
6	rs3077	*HLA-DPA1*	C	T	0.2026	0.7974	Efficacy	3	peginterferon alfa-2b	Hepatitis B, Chronic
6	rs1042151	*HLA-DPB1*	G	A	0.1921	0.8079	Toxicity/ADR	3	aspirin	aspirin-induced asthma,Asthma
6	rs3097671	*HLA-DPB1*	C	G	0.1816	0.8184	Toxicity/ADR	3	aspirin	aspirin-induced asthma
6	rs3129294	*HLA-DPB2*	G	T	0.3132	0.6868	Toxicity/ADR	3	aspirin	aspirin-induced asthma,Asthma
6	rs9272105	*HLA-DQA1*	G	A	0.4368	0.5632	Efficacy	3	interferon beta-1a,interferon beta-1b	

6	rs1063320	*HLA-G*	G	C	0.3	0.7	Efficacy	3	hmg coa reductase inhibitors	Asthma
6	rs2227956	*HSPA1L*	C	T	0.1132	0.8868	Toxicity/ADR	3	carbamazepine	Epilepsy
6	rs6924995	*LOC107986517*	G	A	0.3342	0.6658	Efficacy	4	simvastatin	Hypercholesterolemia
6	rs10945919	*LOC107986666*	G	A	0.2789	0.7211	Efficacy	3	Tumor necrosis factor alpha (TNF-alpha) inhibitors	Arthritis, Rheumatoid
6	rs10455872	*LPA*	G	A	0.03947	0.96053	Efficacy,Toxicity/ADR	2B	hmg coa reductase inhibitors	Coronary Artery Disease
6	rs3828913	*MICB*	A	C	0.03968	0.96032	Efficacy	3	peginterferon alfa-2a,peginterferon alfa-2b,ribavirin	Hepatitis C, Chronic
6	rs10485058	*OPRM1*	G	A	0.07368	0.92632	Efficacy	3	methadone	Opioid-Related Disorders
6	rs1799971	*OPRM1*	G	A	0.1868	0.8132	Efficacy	2B	naloxone	
6	rs9479757	*OPRM1*	A	G	0.07895	0.92105	Other	3	opioids	Opioid-Related Disorders
6	rs3130501	*POU5F1*	A	G	0.2553	0.7447	Toxicity/ADR	3	allopurinol	Epidermal Necrolysis, Toxic,severe cutaneous adverse reactions,Stevens-Johnson Syndrome
6	rs3130931	*POU5F1*	A	G	0.2816	0.7184	Toxicity/ADR	3	allopurinol	Epidermal Necrolysis, Toxic,severe cutaneous adverse reactions,Stevens-Johnson Syndrome
6	rs2016520	*PPARD*	G	A	0.1789	0.8211	Efficacy	3	docetaxel,thalidomide	Prostatic Neoplasms
6	rs2233945	*PSORS1C1*	T	G	0.1368	0.8632	Efficacy	3	etanercept	Arthritis, Rheumatoid
6	rs3131003	*PSORS1C1*	A	G	0.4418	0.5582	Toxicity/ADR	3	allopurinol	severe cutaneous adverse reactions
6	rs3815087	*PSORS1C1*	T	C	0.2158	0.7842	Toxicity/ADR	4	allopurinol	Epidermal Necrolysis, Toxic,severe cutaneous adverse reactions,Stevens-Johnson Syndrome
6	rs9263726	*PSORS1C1*	A	G	0.1158	0.8842	Toxicity/ADR	3	allopurinol	drug reaction with eosinophilia and systemic symptoms,Stevens-Johnson Syndrome

6	rs3094188	*PSORS1C3*	G	T	0.3868	0.6132	Toxicity/ADR	3	allopurinol	Epidermal Necrolysis, Toxic,severe cutaneous adverse reactions,Stevens-Johnson Syndrome
6	rs2282143	*SLC22A1*	T	C	0.05163	0.94837	Metabolism/PK	3	metformin	
6	rs34130495	*SLC22A1*	A	G	0.0079	0.992105	Other	3	tramadol	
6	rs628031	*SLC22A1*	A	G	0.2263	0.7737	Efficacy	3	metformin	Diabetes Mellitus, Type 2
6	rs72552763	*SLC22A1*	D	I	0.3237	0.6763	Metabolism/PK	3	metformin	
6	rs12210538	*SLC22A16*	G	A	0.2105	0.7895	Toxicity/ADR	3	cyclophosphamide,doxorubicin	Breast Neoplasms
6	rs6907567	*SLC22A16*	G	A	0.15	0.85	Efficacy,Toxicity/ADR	3	cyclophosphamide,doxorubicin,fluorouracil	Breast Neoplasms
6	rs316019	*SLC22A2*	T	G	0.06053	0.93947	Toxicity/ADR	3	cisplatin	Neoplasms
6	rs4149178	*SLC22A7*	G	A	0.1667	0.8333	Toxicity/ADR	3	capecitabine	Diarrhea,Neoplasms
6	rs760370	*SLC29A1*	G	A	0.3605	0.6395	Efficacy	3	gemcitabine	Neoplasms
6	rs2071888	*TAPBP*	C	G	0.3968	0.6032	Toxicity/ADR	3	aspirin	Asthma
6	rs2073724	*TCF19*	T	C	0.1026	0.8974	Toxicity/ADR	3	nevirapine	HIV
6	rs1799964	*TNF*	C	T	0.1711	0.8289	Efficacy	3	Tumor necrosis factor alpha (TNF-alpha) inhibitors	Spondylitis, Ankylosing
6	rs6920220	*TNFAIP3*	A	G	0.1085	0.8915	Efficacy	3	methotrexate	
6	rs2500535	*UST*	A	G	0.1868	0.8132	Efficacy	3	nortriptyline	Depressive Disorder, Major
6	rs3025000	*VEGFA*	T	C	0.3237	0.6763	Efficacy	3	bevacizumab,ranibizumab	Macular Degeneration
6	rs3130100	*ZBTB22*	T	C	0.45	0.55	Toxicity/ADR	3	aspirin	aspirin-induced asthma,Asthma
6	rs2647087	-	C	A	0.3553	0.6447	Toxicity/ADR	3	azathioprine,mercaptopurine	Drug Toxicity,Inflammatory Bowel Diseases,Pancreatitis
6	rs2844665	-	A	G	0.2816	0.7184	Toxicity/ADR	3	allopurinol	Epidermal Necrolysis, Toxic,severe cutaneous adverse reactions,Stevens-Johnson Syndrome
6	rs4273729	-	C	G	0.3579	0.6421	Efficacy	3	peginterferon alfa-2a,peginterferon alfa-	Hepatitis C, Chronic

									2b,ribavirin	
7	rs1128503	*ABCB1*	T	C	0.4553	0.5447	Other	3	phenytoin	
7	rs3213619	*ABCB1*	C	T	0.08684	0.91316	Toxicity/ADR	3	atenolol	Hypertension
7	rs9282564	*ABCB1*	G	A	0.02895	0.97105	Toxicity/ADR	3	morphine	
7	rs17143212	*ABCB5*	T	C	0.01316	0.98684	Toxicity/ADR	3	haloperidol	Psychotic Disorders
7	rs4410790	*AHR*	C	T	0.3211	0.6789	Metabolism/PK	3	olanzapine	Psychotic Disorders
7	rs1800111	*CFTR*	C	G	0.02105	0.97895	Efficacy	4	ivacaftor	Cystic Fibrosis
7	rs255100	*CRHR2*	T	A	0.4842	0.5158	Efficacy	3	salbutamol,selective beta-2-adrenoreceptor agonists	Asthma
7	rs342293	*CTB-30L5.1*	G	C	0.4184	0.5816	Efficacy	4	clopidogrel	Coronary Artery Disease
7	rs3735451	*CYP3A4*	G	A	0.2237	0.7763	Toxicity/ADR	3	methadone	Heroin Dependence
7	rs472660	*CYP3A43*	T	C	0.1263	0.8737	Metabolism/PK	3	olanzapine	Schizophrenia
7	rs6977820	*DPP6*	T	C	0.2474	0.7526	Toxicity/ADR	2B	antipsychotics	Schizophrenia,tardive dyskinesia
7	rs712829	*EGFR*	T	G	0.2064	0.7936	Toxicity/ADR	3	erlotinib	Neoplasms
7	rs712830	*EGFR*	A	C	0.2312	0.7688	Toxicity/ADR	3	cetuximab,irinotecan,leucovorin,tegafur	Colorectal Neoplasms
7	rs478437	*LOC105375551*	C	T	0.3711	0.6289	Efficacy	4	tamoxifen	
7	rs4720833	*MAFK*	A	G	0.35	0.65	Toxicity/ADR	3	isoniazid	drug-induced liver injury,Toxic liver disease
7	rs17655652	*NPC1L1*	C	T	0.2868	0.7132	Efficacy	3	pravastatin	Diabetes Mellitus,Hypertension,Vascular Diseases
7	rs854555	*PON1*	A	C	0.4263	0.5737	Efficacy	3	Tumor necrosis factor alpha (TNF-alpha) inhibitors	
7	rs2227631	*SERPINE1*	A	G	0.3895	0.6105	Efficacy	3	antidepressants,citalopram,fluoxetine	Depressive Disorder, Major
7	rs6092	*SERPINE1*	A	G	0.07632	0.92368	Toxicity/ADR	3	dexamethasone	Precursor Cell Lymphoblastic Leukemia-Lymphoma
7	rs4379368	*SUGCT*	T	C	0.08947	0.91053	Toxicity/ADR	3	hormonal contraceptives for	

									systemic use	
7	rs846664	*TAS2R16*	G	T	0.01852	0.98148	Toxicity/ADR	3	ethanol	Alcoholism
7	rs2960436	-	A	G	0.4108	0.5892	Efficacy	3	fluorouracil	Stomach Neoplasms
7	rs34548976	-	T	C	0.3632	0.6368	Efficacy	3	adrenergics, inhalants	Asthma
7	rs6966038	-	G	A	0.1395	0.8605	Efficacy	4	citalopram	Depressive Disorder, Major
8	rs1517114	*C8orf34*	C	G	0.3842	0.6158	Toxicity/ADR	2B	irinotecan	Carcinoma, Non-Small-Cell Lung
8	rs6983267	*CCAT2, CASC8*	T	G	0.4132	0.5868	Efficacy	3	Platinum compounds	Lung Neoplasms
8	rs6988229	*COL22A1*	T	C	0.1553	0.8447	Efficacy	2B	salbutamol	Asthma
8	rs7387065	*CSMD1*	G	A	0.2921	0.7079	Efficacy	3	hydrochlorothiazide	Essential hypertension
8	rs1799998	*CYP11B2*	C	T	0.4471	0.5529	Efficacy	3	benazepril,imidapril	Essential hypertension
8	rs2669429	*DPYS*	C	T	0.4579	0.5421	Toxicity/ADR	3	atenolol	Hyperglycemia,Hypertension
8	rs352428	*EXTL3*	A	G	0.1026	0.8974	Efficacy	3	citalopram,escitalopram	Depressive Disorder, Major
8	rs13253389	*NAT1*	A	G	0.45	0.55	Metabolism/PK	3	cotinine	
8	rs1041983	*NAT2*	T	C	0.3132	0.6868	Toxicity/ADR	2A	ethambutol,isoniazid,pyrazinamide,rifampin	Tuberculosis
8	rs1208	*NAT2*	G	A	0.3421	0.6579	Toxicity/ADR	3	isoniazid,phenytoin	Drug interaction with drug,Drug Toxicity
8	rs1799929	*NAT2*	T	C	0.3395	0.6605	Toxicity/ADR	3	Drugs For Treatment Of Tuberculosis	Tuberculosis
8	rs1799930	*NAT2*	A	G	0.1553	0.8447	Toxicity/ADR,Metabolism/PK	2A	ethambutol,isoniazid,pyrazinamide,rifampin	Tuberculosis
8	rs1799931	*NAT2*	A	G	0.15	0.85	Toxicity/ADR	3	docetaxel,thalidomide	Prostatic Neoplasms
8	rs1801280	*NAT2*	C	T	0.3396	0.6604	Toxicity/ADR	3	cisplatin,cyclophosphamide	Ovarian Neoplasms
8	rs17060812	*SLC39A14*	T	C	0.07105	0.92895	Efficacy	3	nortriptyline	Depression
8	rs1495741	-	G	A	0.3316	0.6684	Toxicity/ADR	3	Drugs For Treatment Of Tuberculosis,isoniazid	Tuberculosis
9	rs2230808	*ABCA1*	A	G	0.2237	0.7763	Efficacy	3	fenofibrate	Hypertriglyceridemia
9	rs495828	*ABO*	T	G	0.2026	0.7974	Toxicity/ADR	3	Ace Inhibitors, Plain	

9	rs8176719	*ABO*	I	D	0.3105	0.6895	Toxicity/ADR	3	hormonal contraceptives for systemic use	
9	rs3849942	*C9orf72*	A	G	0.1421	0.8579	Efficacy	4	Tumor necrosis factor alpha (TNF-alpha) inhibitors	Arthritis, Rheumatoid
9	rs774359	*C9orf72*	C	T	0.1579	0.8421	Efficacy	3	Tumor necrosis factor alpha (TNF-alpha) inhibitors	Arthritis, Rheumatoid
9	rs10757274	*CDKN2B-AS1*	A	G	0.4974	0.5026	Toxicity/ADR	3	Antiinflammatory agents, non-steroids	Acute coronary syndrome
9	rs11141915	*DAPK1*	C	A	0.1941	0.8059	Toxicity/ADR	4	gemcitabine	Neoplasms
9	rs1611131	*DBH*	G	A	0.3632	0.6368	Toxicity/ADR	3	opioids	Opioid-Related Disorders
9	rs430397	*HSPA5*	A	G	0.05	0.95	Efficacy	3	Platinum compounds	Carcinoma, Non-Small-Cell Lung
9	rs2814707	*MOB3B*	A	G	0.1402	0.8598	Efficacy	3	Tumor necrosis factor alpha (TNF-alpha) inhibitors	Arthritis, Rheumatoid
9	rs7046653	*MOB3B*	A	G	0.3474	0.6526	Efficacy	3	Tumor necrosis factor alpha (TNF-alpha) inhibitors	Arthritis, Rheumatoid
9	rs2289658	*NTRK2*	G	A	0.1632	0.8368	Dosage	3	methadone	Heroin Dependence,Opioid-Related Disorders
9	rs2378676	*NTRK2*	T	G	0.4605	0.5395	Dosage	3	methadone	Heroin Dependence,Opioid-Related Disorders
9	rs10306114	*PTGS1*	G	A	0.04737	0.95263	Efficacy	2B	aspirin	Coronary Artery Disease,Myocardial Infarction
9	rs301435	*SLC1A1*	A	G	0.2711	0.7289	Efficacy	4	Selective serotonin reuptake inhibitors	Obsessive-Compulsive Disorder
9	rs4986790	*TLR4*	G	A	0.02895	0.97105	Efficacy	3	pravastatin	Coronary Artery Disease
9	rs3761847	*TRAF1*	G	A	0.2921	0.7079	Efficacy	3	adalimumab,etanercept,infliximab,Tumor necrosis factor alpha (TNF-alpha) inhibitors	Arthritis, Rheumatoid
9	rs7862221	*TSC1*	C	T	0.1211	0.8789	Toxicity/ADR	3	aspirin	aspirin-induced asthma,Asthma
9	rs1800975	*XPA*	A	G	0.2763	0.7237	Efficacy	3	Platinum compounds	Carcinoma, Non-Small-Cell Lung
9	rs10811661	-	C	T	0.1402	0.8598	Efficacy	3	troglitazone	Diabetes Mellitus, Type 2

10	rs3740065	*ABCC2*	C	T	0.1053	0.8947	Toxicity/ADR,Metabolism/PK	3	methotrexate	Leukemia, B-Cell, Acute
10	rs717620	*ABCC2*	A	G	0.1289	0.8711	Other	3	erythromycin	
10	rs1800545	*ADRA2A*	A	G	0.04474	0.95526	Efficacy	3	atenolol	Hypertrophy, Left Ventricular
10	rs10994982	*ARID5B*	G	A	0.4368	0.5632	Metabolism/PK	3	methotrexate	Precursor Cell Lymphoblastic Leukemia-Lymphoma
10	rs4948496	*ARID5B*	T	C	0.3447	0.6553	Metabolism/PK	3	methotrexate	Precursor Cell Lymphoblastic Leukemia-Lymphoma
10	rs10490924	*ARMS2*	T	G	0.2658	0.7342	Efficacy	3	bevacizumab	Macular Degeneration
10	rs12415607	*CASP7*	A	C	0.3395	0.6605	Efficacy	3	docetaxel,gemcitabine,paclitaxel,Platinum compounds,vinorelbine	Carcinoma, Non-Small-Cell Lung
10	rs7921977	*CASP7*	C	T	0.3211	0.6789	Efficacy	3	docetaxel,gemcitabine,paclitaxel,Platinum compounds,vinorelbine	Carcinoma, Non-Small-Cell Lung
10	rs3810950	*CHAT*	A	G	0.1605	0.8395	Efficacy	4	olanzapine	Schizophrenia
10	rs10997242	*CTNNA3*	C	T	0.1684	0.8316	Efficacy	3	antidepressants	Depressive Disorder, Major
10	rs11188072	*CYP2C19*	T	C	0.1587	0.8413	Dosage	3	mephenytoin	
10	rs10509681	*CYP2C8*	C	T	0.06053	0.93947	Metabolism/PK	2A	rosiglitazone	
10	rs11572080	*CYP2C8*	A	G	0.05615	0.94385	Dosage	3	repaglinide	
10	rs10509680	*CYP2C9*	T	G	0.03947	0.96053	Dosage	3	warfarin	
10	rs4086116	*CYP2C9*	T	C	0.09474	0.90526	Dosage	3	acenocoumarol	
10	rs2031920	*CYP2E1*	T	C	0.1277	0.8723	Toxicity/ADR	3	Drugs For Treatment Of Tuberculosis	Tuberculosis
10	rs2289310	*DLG5*	A	C	0.05526	0.94474	Efficacy	4	capecitabine,fluorouracil	Neoplasm Metastasis
10	rs3824662	*GATA3*	T	G	0.3553	0.6447	Efficacy	3	asparaginase,cyclophosphamide,cytarabine,daunorubicin,dexamethasone,doxorubicin,leucovorin,mercaptopurine,methotrexate,pegaspargase,prednisone,thioguanine,vincristine	

10	rs10509373	*LRMDA*	C	T	0.4153	0.5847	Efficacy	3	tamoxifen	Breast Neoplasms
10	rs4933824	*NRG3*	T	G	0.03158	0.96842	Toxicity/ADR	3	iloperidone	Acquired Long QT Syndrome (aLQTS)
10	rs11189381	*SFRP5*	C	T	0.1526	0.8474	Toxicity/ADR	3	Bisphosphonates	Osteonecrosis
10	rs7903146	*TCF7L2*	T	C	0.2395	0.7605	Efficacy	2B	sulfonamides, urea derivatives	
10	rs11252394	-	A	G	0.03684	0.96316	Efficacy	3	salbutamol	Asthma
10	rs12777823	-	A	G	0.1053	0.8947	Dosage	1A	warfarin	
10	rs7912580	-	A	G	0.1105	0.8895	Efficacy	4	olanzapine,quetiapine,risperidone,ziprasidone	Schizophrenia
11	rs7118900	*ANKK1*	A	G	0.2763	0.7237	Dosage	3	methadone	Heroin Dependence,Opioid-Related Disorders
11	rs662799	*APOA5*	G	A	0.1868	0.8132	Efficacy	2B	atorvastatin,lovastatin,simvastatin	Hyperlipidemias
11	rs1801516	*ATM*	A	G	0.09211	0.90789	Toxicity/ADR	3	cyclophosphamide,doxorubicin,fluorouracil	Breast Neoplasms
11	rs11030104	*BDNF*	G	A	0.1711	0.8289	Efficacy	3	antipsychotics	Schizophrenia
11	rs6265	*BDNF*	A	G	0.1632	0.8368	Efficacy	3	paroxetine	Depressive Disorder, Major
11	rs7103411	*BDNF*	C	T	0.1737	0.8263	Efficacy	3	citalopram	Depressive Disorder, Major
11	rs11212617	*C11orf65*	C	A	0.3737	0.6263	Efficacy	2B	metformin	Diabetes Mellitus, Type 2
11	rs9344	*CCND1*	A	G	0.3684	0.6316	Efficacy	3	cetuximab	Colorectal Neoplasms
11	rs10741657	*CYP2R1*	A	G	0.3737	0.6263	Efficacy	3	peginterferon alfa-2b,ribavirin	Hepatitis C, Chronic
11	rs716274	*DYNC2H1*	G	A	0.4289	0.5711	Toxicity/ADR	2B	etoposide,Platinum compounds	
11	rs1799963	*F2*	A	G	0.01579	0.98421	Toxicity/ADR	3	hormonal contraceptives for systemic use	Stroke,Venous Thrombosis
11	rs61734430	*FOLR3*	T	C	0.01579	0.98421	Efficacy	3	pemetrexed	Carcinoma, Non-Small-Cell Lung,Mesothelioma
11	rs3758785	*GPR83*	G	A	0.2842	0.7158	Efficacy	3	hydrochlorothiazide	Essential hypertension
11	rs1138272	*GSTP1*	T	C	0.01842	0.98158		3	thiotepa	Neoplasms

11	rs1695	*GSTP1*	G	A	0.3868	0.6132	Toxicity/ADR	2A	Platinum compounds	Neoplasms
11	rs1062613	*HTR3A*	T	C	0.2026	0.7974	Efficacy	3	clozapine	Schizophrenia
11	rs11600347	*KCNJ1*	A	C	0.08684	0.91316	Toxicity/ADR	3	hydrochlorothiazide	Hypertension
11	rs5219	*KCNJ11*	T	C	0.4237	0.5763	Efficacy	2A	glibenclamide,gliclazide,glimepiride,glipizide,gliquidone,sulfonamides, urea derivatives	Diabetes Mellitus
11	rs2237892	*KCNQ1*	T	C	0.3079	0.6921	Efficacy	3	repaglinide	Diabetes Mellitus, Type 2
11	rs2237895	*KCNQ1*	C	A	0.4079	0.5921	Efficacy	3	repaglinide	Diabetes Mellitus, Type 2
11	rs619586	*MALAT1*	G	A	0.05789	0.94211	Efficacy	3	Platinum compounds	Lung Neoplasms
11	rs9937	*RRM1*	G	A	0.457	0.543	Efficacy,Toxicity/ADR	3	gemcitabine	Neoplasms
11	rs2306168	*SLCO2B1*	T	C	0.06316	0.93684	Metabolism/PK	3	fexofenadine	
12	rs7968606	*ANKS1B*	T	C	0.07368	0.92632	Efficacy	3	amisulpride	Schizophrenia
12	rs1006737	*CACNA1C*	A	G	0.25	0.75	Toxicity/ADR	4	citalopram	Depressive Disorder, Major
12	rs2270777	*CDK4*	A	G	0.2684	0.7316	Other	3	somatropin recombinant	
12	rs310786	*E2F7*	C	T	0.2395	0.7605	Toxicity/ADR	3	tamoxifen	Breast Neoplasms
12	rs10846744	*SCARB1*	C	G	0.2237	0.7763	Efficacy	3	peginterferon alfa-2a,ribavirin	Hepatitis C, Chronic
12	rs10841753	*SLCO1B1*	C	T	0.2816	0.7184	Metabolism/PK	3	methotrexate	Precursor Cell Lymphoblastic Leukemia-Lymphoma
12	rs11045819	*SLCO1B1*	A	C	0.09474	0.90526	Efficacy	3	fluvastatin	Hypercholesterolemia
12	rs11045879	*SLCO1B1*	C	T	0.1526	0.8474	Toxicity/ADR	2A	methotrexate	Precursor Cell Lymphoblastic Leukemia-Lymphoma
12	rs4149032	*SLCO1B1*	T	C	0.4	0.6	Metabolism/PK	3	rifampin	Tuberculosis
12	rs4149081	*SLCO1B1*	A	G	0.1566	0.8434	Toxicity/ADR	3	methotrexate	Precursor Cell Lymphoblastic Leukemia-Lymphoma
12	rs4363657	*SLCO1B1*	C	T	0.1474	0.8526	Toxicity/ADR	3	simvastatin	Muscular Diseases
12	rs4149117	*SLCO1B3*	T	G	0.1763	0.8237	Toxicity/ADR	3	carboplatin,paclitaxel	Lung Neoplasms
12	rs7311358	*SLCO1B3*	G	A	0.1729	0.8271	Other	3	mycophenolate mofetil	Kidney Transplantation

12	rs3794271	*SLCO1C1*	C	T	0.4947	0.5053	Efficacy	3	etanercept,infliximab,Tumor necrosis factor alpha (TNF-alpha) inhibitors	Arthritis, Psoriatic,Arthritis, Rheumatoid
12	rs4149570	*TNFRSF1A*	T	G	0.336	0.664	Efficacy	3	Tumor necrosis factor alpha (TNF-alpha) inhibitors	Crohn Disease,Inflammatory Bowel Diseases
12	rs4516035	*VDR*	C	T	0.3342	0.6658	Metabolism/PK	4	midazolam	
12	rs7297610	*YEATS4*	T	C	0.05263	0.94737	Efficacy	2B	hydrochlorothiazide	Essential hypertension,Hypertension
12	rs2965667	-	A	T	0.01977	0.98023	Other	3	Antiinflammatory agents, non-steroids,aspirin	Colorectal Neoplasms
13	rs16950650	*ABCC4*	T	C	0.01316	0.98684	Efficacy	3	cisplatin,irinotecan	Carcinoma, Small Cell
13	rs1751034	*ABCC4*	C	T	0.2526	0.7474	Metabolism/PK	2B	tenofovir	HIV,HIV Infections
13	rs77876672	*DIAPH3*	T	C	0.01316	0.98684	Efficacy	3	hydrochlorothiazide	Essential hypertension
14	rs1130214	*AKT1*	T	G	0.2318	0.7682	Efficacy	3	carboplatin,cisplatin	Lung Neoplasms
14	rs12050217	*BDKRB1*	G	A	0.2947	0.7053	Efficacy	3	perindopril	Coronary Artery Disease
14	rs8012552	*BDKRB2*	T	C	0.4921	0.5079	Toxicity/ADR	3	Ace Inhibitors, Plain	Cough,Hypertension
14	rs4982133	*EGLN3-AS1*, *LOC102724945*	A	C	0.15	0.85	Efficacy	4	methotrexate	Arthritis, Rheumatoid
14	rs2498804	*LOC102723342*, *LOC107987209*	T	G	0.4206	0.5794	Efficacy	3	carboplatin,cisplatin	Carcinoma, Non-Small-Cell Lung
14	rs1160351	*MDGA2*	G	T	0.2421	0.7579	Toxicity/ADR	3	fluvoxamine,milnacipran,paroxe tine	Depressive Disorder, Major
14	rs7142881	*NUBPL*	G	A	0.4711	0.5289	Toxicity/ADR	3	iloperidone	Acquired Long QT Syndrome (aLQTS)
14	rs17091162	*SERPINA3*	A	C	0.2447	0.7553	Efficacy	3	antineoplastic agents	Pancreatic Neoplasms
15	rs4646	*CYP19A1*	A	C	0.4237	0.5763	Efficacy	2B	anastrozole,cyclophosphamide,d ocetaxel,doxorubicin,epirubicin, exemestane,fluorouracil,paclitax el,radiotherapy,tamoxifen	Breast Neoplasms,Menopause
15	rs700518	*CYP19A1*	G	A	0.3605	0.6395	Other	3	letrozole	Breast Neoplasms,Menopause

15	rs2472304	*CYP1A2*	A	G	0.3447	0.6553	Efficacy	3	paroxetine	Depressive Disorder, Major
15	rs762551	*CYP1A2*	C	A	0.2342	0.7658	Toxicity/ADR	3	leflunomide	Arthritis, Rheumatoid
15	rs2412459	*EIF2AK4*	C	T	0.1289	0.8711	Efficacy	4	olanzapine,quetiapine,risperidone,ziprasidone	Schizophrenia
15	rs1800588	*LIPC*	T	C	0.3868	0.6132	Efficacy	3	fluvastatin,simvastatin	
15	rs16973225	*LOC102724001*	C	A	0.06579	0.93421	Other	3	Antiinflammatory agents, non-steroids,aspirin	Colorectal Neoplasms
15	rs2290271	*SLC28A1*	G	T	0.4553	0.5447	Toxicity/ADR	3	anthracyclines and related substances	
15	rs1719247		C	T	0.4395	0.5605	Toxicity/ADR	2B	hmg coa reductase inhibitors,simvastatin	
16	rs119774	*ABCC1*	A	G	0.05263	0.94737	Efficacy	3	montelukast	Asthma
16	rs45511401	*ABCC1*	T	G	0.03158	0.96842	Toxicity/ADR	3	doxorubicin	Arrhythmias, Cardiac,Drug Toxicity,Lymphoma, Non-Hodgkin
16	rs2238472	*ABCC6*	A	G	0.3658	0.6342	Toxicity/ADR	3	docetaxel,thalidomide	Prostatic Neoplasms
16	rs1967309	*ADCY9*	C	T	0.4605	0.5395	Efficacy,Toxicity/ADR	3	dalcetrapib	Acute coronary syndrome
16	rs8192935	*CES1*	T	C	0.5	0.5	Metabolism/PK	3	Dabigatran	Atrial Fibrillation
16	rs3785161	*CES1P1*	C	A	0.2566	0.7434	Efficacy	3	clopidogrel	Coronary Disease
16	rs1532624	*CETP*	T	G	0.3237	0.6763	Efficacy	2B	hmg coa reductase inhibitors	Hyperlipidemias
16	rs3213422	*DHODH*	A	C	0.3605	0.6395	Efficacy	4	leflunomide	Arthritis, Rheumatoid
16	rs10782001	*FBXL19*	G	A	0.3711	0.6289	Toxicity/ADR	3	Tumor necrosis factor alpha (TNF-alpha) inhibitors	Psoriasis
16	rs9939609	*FTO*	A	T	0.2526	0.7474	Efficacy	3	interferon alfa-2a, recombinant,interferon alfa-2b, recombinant,ribavirin	Hepatitis C, Chronic,HIV
16	rs2232228	*HAS3*	G	A	0.45	0.55	Toxicity/ADR	2B	anthracyclines and related substances	Cardiomyopathies
16	rs2066844	*NOD2*	T	C	0.03743	0.96257	Efficacy	3	tacrolimus	Kidney Transplantation

16	rs1800566	*NQO1*	T	C	0.3842	0.6158	Efficacy	2A	Alkylating Agents,anthracyclines and related substances,fluorouracil,Platinum compounds	Breast Neoplasms,Carcinoma, Non-Small-Cell Lung,Neoplasms,Ovarian Neoplasms,Stomach Neoplasms
16	rs3888190	*SH2B1*	G	T	0.4674	0.5326	Toxicity/ADR	3	amisulpride,aripiprazole,clozapine,lithium,mirtazapine,olanzapine,paliperidone,quetiapine,risperidone,valproic acid	Bipolar Disorder,Depressive Disorder,Psychotic Disorders,schizoaffective disorder
16	rs10871454	*STX4*	T	C	0.4921	0.5079	Dosage	3	warfarin	
16	rs11150606	*VKORC1*	C	T	0.2684	0.7316		3	warfarin	
16	rs17708472	*VKORC1*	A	G	0.1605	0.8395	Dosage	2A	warfarin	
16	rs2359612	*VKORC1*	A	G	0.4974	0.5026	Dosage	2A	warfarin	
16	rs2884737	*VKORC1*	C	A	0.1658	0.8342	Dosage	2A	warfarin	
16	rs7294	*VKORC1*	A	G	0.3182	0.6818	Dosage	1B	warfarin	
16	rs8050894	*VKORC1*	C	G	0.4974	0.5026	Dosage	2A	warfarin	
16	rs247616	-	T	C	0.2632	0.7368	Efficacy	3	hmg coa reductase inhibitors	
16	rs7186128	-	G	A	0.3421	0.6579	Efficacy	3	cisplatin,irinotecan	
16	rs7405404	-	T	C	0.1905	0.8095	Efficacy	3	lithium	Bipolar Disorder
16	rs9936750	-	C	T	0.09737	0.90263	Toxicity/ADR	3	capecitabine	Neoplasms
17	rs4343	*ACE*	G	A	0.4418	0.5582	Efficacy	3	sildenafil	Erectile Dysfunction
17	rs4790694	*ARRB2*	A	C	0.1553	0.8447	Toxicity/ADR	3	methamphetamine	Substance-Related Disorders
17	rs4541111	*AXIN2*	G	T	0.4947	0.5053	Efficacy	3	Platinum compounds	Carcinoma, Non-Small-Cell Lung
17	rs9901675	*CD68*	A	G	0.01579	0.98421	Efficacy	4	methylphenidate	Attention Deficit Disorder with Hyperactivity
17	rs4562	*CLDN7, ELP5*	A	G	0.4465	0.5535	Efficacy	4	methylphenidate	Attention Deficit Disorder with Hyperactivity
17	rs6065	*GP1BA*	T	C	0.1316	0.8684	Efficacy	2B	aspirin	
17	rs5918	*ITGB3*	C	T	0.08158	0.91842	Efficacy	3	aspirin	Acute coronary syndrome,Coronary Artery

										Disease
17	rs1052536	*LIG3*	T	C	0.3789	0.6211	Toxicity/ADR	3	cisplatin,cyclophosphamide	Ovarian Neoplasms
17	rs1000940	*RABEP1*	C	T	0.4079	0.5921	Other	3	amisulpride,aripiprazole,clozapine,lithium,mirtazapine,olanzapine,paliperidone,quetiapine,risperidone,valproic acid	Bipolar Disorder,Depressive Disorder,Psychotic Disorders,schizoaffective disorder
17	rs11552708	*SENP3*	A	G	0.1164	0.8836	Efficacy	4	methylphenidate	Attention Deficit Disorder with Hyperactivity
17	rs1979277	*SHMT1*	A	G	0.2593	0.7407	Toxicity/ADR	3	methotrexate	Precursor Cell Lymphoblastic Leukemia-Lymphoma
17	rs12943590	*SLC47A2*	A	G	0.4497	0.5503	Metabolism/PK	3	metformin	
17	rs11868035	*SREBF1*	A	G	0.4737	0.5263	Toxicity/ADR	3	hmg coa reductase inhibitors	Schizophrenia
17	rs4796793	*STAT3*	G	C	0.1658	0.8342	Efficacy	3	interferons	Carcinoma, Renal Cell
17	rs1042522	*TP53*	C	G	0.2132	0.7868	Efficacy,Toxicity/ADR	2B	antineoplastic agents	Neoplasms
17	rs4267385	-	T	C	0.45	0.55	Toxicity/ADR	3	Ace Inhibitors, Plain	Cough
17	rs879207	-	G	A	0.2895	0.7105	Toxicity/ADR	3	carboplatin,docetaxel,paclitaxel	Ovarian Neoplasms
18	rs4799915	*CELF4*	C	T	0.3053	0.6947	Toxicity/ADR	3	iloperidone	Acquired Long QT Syndrome (aLQTS)
18	rs2612091	*ENOSF1*	G	A	0.4368	0.5632	Toxicity/ADR	3	capecitabine	Neoplasms
18	rs17782313	*MC4R*	C	T	0.1184	0.8816	Toxicity/ADR	2B	antipsychotics	Schizophrenia
18	rs489693	*MC4R*	A	C	0.2579	0.7421	Toxicity/ADR	2B	amisulpride,aripiprazole,clozapine,haloperidol,olanzapine,paliperidone,quetiapine,risperidone,ziprasidone	Autism Spectrum Disorder,schizoaffective disorder,Schizophrenia
18	rs4149601	*NEDD4L*	A	G	0.1789	0.8211	Efficacy	2B	diuretics,hydrochlorothiazide	Hypertension
18	rs6506569	*PTPRM*	T	C	0.4316	0.5684	Efficacy	4	methotrexate	Arthritis, Rheumatoid
18	rs1805034	*TNFRSF11A*	C	T	0.4078	0.5922	Toxicity/ADR	3	acetaminophen,aspirin,diclofenac,propionic acid derivatives,Pyrazolones	
18	rs2853741	*TYMS*	T	C	0.2579	0.7421	Toxicity/ADR	3	capecitabine	Diarrhea,Neoplasms

19	rs445925	*APOC1*	T	C	0.06053	0.93947	Efficacy	3	hmg coa reductase inhibitors	
19	rs3213239	*APLF*	D	I	0.2447	0.7553	Efficacy	3	Platinum compounds	Carcinoma, Non-Small-Cell Lung
19	rs7412	*APOC1, APOE*	T	C	0.04497	0.95503	Toxicity/ADR	3	ritonavir	HIV,HIV Infections,Hyperlipidemias,Hypertriglyceridemia
19	rs56113850	*CYP2A6*	T	C	0.3641	0.6359	Metabolism/PK	3	nicotine	Tobacco Use Disorder
19	rs3786547	*CYP2B6*	C	T	0.3369	0.6631	Toxicity/ADR	3	nevirapine	HIV
19	rs13181	*ERCC2*	G	T	0.2237	0.7763	Efficacy,Toxicity/ADR	3	fluorouracil,leucovorin,oxaliplatin	Colorectal Neoplasms
19	rs1613662	*GP6*	G	A	0.07895	0.92105	Efficacy	3	aspirin	Coronary Artery Disease
19	rs12980275	*IFNL3*	G	A	0.3684	0.6316	Efficacy	3	peginterferon alfa-2a,peginterferon alfa-2b,ribavirin	Hepatitis C, Chronic
19	rs2562456	*LINC00664*	G	A	0.4026	0.5974	Efficacy	3	ketorolac	
19	rs3814995	*NPHS1*	T	C	0.3816	0.6184	Efficacy	3	losartan	Hypertension
19	rs1126510	*PTGIR*	C	T	0.1763	0.8237	Toxicity/ADR	3	aspirin	Asthma
19	rs1800469	*TGFB1*	T	C	0.4342	0.5658	Toxicity/ADR	3	aspirin	
19	rs1800471	*TGFB1*	C	G	0.03684	0.96316	Efficacy	3	rituximab	Arthritis, Rheumatoid
19	rs10420097	*ZNF211*	G	A	0.04233	0.95767	Efficacy	4	methylphenidate	Attention Deficit Disorder with Hyperactivity
20	rs6138150	*CST5*	C	T	0.2842	0.7158	Efficacy	3	Tumor necrosis factor alpha (TNF-alpha) inhibitors	Arthritis, Rheumatoid
20	rs2248359	*CYP24A1*	T	C	0.3132	0.6868	Metabolism/PK	3	deferasirox	beta-Thalassemia
20	rs7270101	*ITPA*	C	A	0.09211	0.90789	Toxicity/ADR	2B	azathioprine	Inflammatory Bowel Diseases,Precursor Cell Lymphoblastic Leukemia-Lymphoma
20	rs6028945	*MAFB*	T	G	0.08947	0.91053	Efficacy	3	Tumor necrosis factor alpha (TNF-alpha) inhibitors	Arthritis, Rheumatoid
20	rs2273359	*NELFCD*	G	C	0.03684	0.96316	Efficacy	4	diuretics,hydrochlorothiazide,Th	Hypertension

									iazides, plain	
20	rs6021191	*NFATC2*	T	A	0.07105	0.92895	Toxicity/ADR	3	asparaginase	Precursor Cell Lymphoblastic Leukemia-Lymphoma
20	rs6127921	-	C	A	0.2	0.8	Efficacy	4	citalopram	Depressive Disorder, Major
21	rs9981861	*DSCAM*	C	T	0.35	0.65	Efficacy	3	carboplatin,paclitaxel	Carcinoma, Non-Small-Cell Lung,Neoplasms
21	rs2832407	*GRIK1*	C	A	0.4868	0.5132	Efficacy,Metabolism/PK	3	topiramate	Alcohol-Related Disorders
22	rs2071421	*ARSA*	G	A	0.3	0.7	Efficacy	4	methylphenidate	Attention Deficit Disorder with Hyperactivity
22	rs4680	*COMT*	A	G	0.3862	0.6138	Efficacy	2A	nicotine	Tobacco Use Disorder
22	rs5993883	*COMT*	G	T	0.4105	0.5895	Efficacy	3	quetiapine	Schizophrenia
22	rs738409	*PNPLA3*	G	C	0.4211	0.5789	Toxicity/ADR	2B	asparaginase,cyclophosphamide, daunorubicin,prednisolone,vincristine	Precursor Cell Lymphoblastic Leukemia-Lymphoma
22	rs4823613	*PPARA*	G	A	0.3368	0.6632	Efficacy	3	simvastatin	
22	rs11479	*TYMP*	T	C	0.1553	0.8447	Toxicity/ADR	3	capecitabine,fluorouracil	Neoplasms
22	rs518350	-	A	G	0.07937	0.92063	Efficacy	3	salbutamol	Asthma

Abbreviations: A1, minor allele nucleotide; A2, major allele nucleotide; AF, allele frequency; CHR, chromosome; SNP, single nucleotide polymorphism.

Table S2. The frequencies of Class I and II HLA-associated drug hypersensitivity and related drug adverse reactions in the Chilean population.

HLA pharmacogenetic marker	AF Chilean	Drug/Molecule	Phenotype Categories
*HLA-A*01:01*	10.00	Phenobarbital	Toxicity
*HLA-A*02:01*	23.95	Allopurinol	Toxicity
*HLA-A*11:01*	5.00	Levetiracetam	Toxicity
		Carbamazepine	Toxicity
*HLA-A*24:02*	9.21	Carbamazepine	Toxicity
		Phenytoin	Toxicity
		Lamotrigine	Toxicity
		Dapsone	Toxicity
*HLA-A*30:02*	1.84	Amoxicillin-Clavulanate	Toxicity
*HLA-A*31:01*	5.53	Carbamazepine	Toxicity
		Lamotrigine	Toxicity
		Oxcarbazepine	Toxicity
*HLA-A*32:01*	2.89	Carbamazepine	Toxicity
		Vancomycin	Toxicity
*HLA-A*33:03*	0.26	Allopurinol	Toxicity
		Lamotrigine	Toxicity
		Ticlopidine	Toxicity
*HLA-A*68:01*	11.84	Lamotrigine	Toxicity
*HLA-B*07:02*	6.05	Sulfamethoxazole-Trimethoprim	Toxicity
*HLA-B*08:01*	6.32	Infliximab	Toxicity
		Antithyroid Preparations	Toxicity
*HLA-B*13:02*	1.58	Nevirapine	Toxicity
		Oxcarbazepine	Toxicity
*HLA-B*15:01*	1.58	Nevirapine	Toxicity
		Oxcarbazepine	Toxicity
		Interferon beta-1a	Efficacy
*HLA-B*18:01*	5.53	Amoxicillin-Clavulanate	Toxicity
*HLA-B*35:05*	0.26	Nevirapine	Toxicity
		Minocycline	Toxicity

*HLA-B*38:01*	2.63	Co-trimoxazole	Toxicity
*HLA-B*39:05*	4.74	Nevirapine	Toxicity
*HLA-B*39:06*	0.26	Nevirapine	Toxicity
*HLA-B*39:09*	9.74	Nevirapine	Toxicity
*HLA-B*40:01*	1.58	Carbamazepine	Toxicity
		Oxcarbazepine	Toxicity
*HLA-B*40:02*	2.11	Oxcarbazepine	Toxicity
*HLA-B*44:02*	3.68	Interferon alfa-2a, recombinant, ribavirin	Efficacy
*HLA-B*44:03*	5.53	Ticlopidine	Toxicity
		Lamotrigine	Toxicity
*HLA-B*48:01*	2.11	Allopurinol	Toxicity
*HLA-B*51:01*	7.89	Nevirapine	Toxicity
		Carbamazepine	Toxicity
		Clindamycin	Toxicity
		Phenobarbital	Toxicity
*HLA-B*52:01*	0.53	Nevirapine	Toxicity
*HLA-B*55:01*	1.32	Nevirapine	Toxicity
		Penicillin g, Penicillin v	Toxicity
*HLA-B*56:01*	0.26	Nevirapine	Toxicity
*HLA-B*57:01*	1.58	Abacavir	Toxicity
		Flucloxacillin	Toxicity
		Nevirapine	Toxicity
*HLA-B*58:01*	0.53	Allopurinol	Toxicity
		Lamotrigine	Toxicity
		Antiepileptics	Toxicity
		Carbamazepine	Toxicity
*HLA-C*01:02*	5.00	Methazolamide	Toxicity
		Peginterferon alfa-2b, Ribavirin	Efficacy
*HLA-C*02:02*	3.68	Peginterferon alfa-2b, Ribavirin	Efficacy
*HLA-C*04:01*	12.63	Nevirapine	Toxicity
		Peginterferon alfa-2b, Ribavirin	Efficacy
*HLA-C*05:01*	5.79	Nevirapine	Toxicity

		Peginterferon alfa-2b, Ribavirin	Efficacy
*HLA-C*06:02*	7.89	Co-trimoxazole	Toxicity
		Sulfamethoxazole-Trimethoprim	Toxicity
		Ustekinumab	Efficacy
		Methotrexate	Efficacy
		Peginterferon alfa-2b, Ribavirin	Efficacy
*HLA-C*07:01*	22.37	Peginterferon alfa-2b, Ribavirin	Efficacy
*HLA-C*07:02*	1.05	Sulfamethoxazole-Trimethoprim	Toxicity
*HLA-C*08:01*	1.05	Carbamazepine	Toxicity
		Phenytoin	Toxicity
		Allopurinol	Toxicity
		Sulfamethoxazole-Trimethoprim	Toxicity
		Peginterferon alfa-2b, Ribavirin	Efficacy
*HLA-C*12:02*	0.26	Peginterferon alfa-2b, Ribavirin	Efficacy
*HLA-C*12:03*	4.21	Infliximab	Toxicity
*HLA-C*14:02*	0.79	Peginterferon alfa-2b, Ribavirin	Efficacy
*HLA-C*15:02*	6.84	Peginterferon alfa-2b, Ribavirin	Efficacy
*HLA-C*16:01*	4.21	Peginterferon alfa-2b, Ribavirin	Efficacy
*HLA-C*17:01*	1.32	Peginterferon alfa-2b, Ribavirin	Efficacy
*HLA-DRB1*01:01*	3.16	Nevirapine	Toxicity
		Atorvastatin, Fluvastatin, HMG-CoA Reductase Inhibitors, Pravastatin, Rosuvastatin, Simvastatin	Toxicity
*HLA-DRB1*01:02*	2.89	Nevirapine	Toxicity
*HLA-DRB1*01:03*	0.79	Nevirapine	Toxicity
*HLA-DRB1*03:01*	10.26	Infliximab	Toxicity
		Allopurinol	Toxicity
*HLA-DRB1*04:01*	1.05	Interferon beta-1a	Efficacy
*HLA-DRB1*04:04*	1.58	Infliximab	Toxicity
		Nevirapine	Toxicity
*HLA-DRB1*07:01*	11.05	Pegaspargase	Toxicity
		Lapatinib	Toxicity

		Azathioprine-Mercaptopurine	Toxicity
*HLA-DRB1*08:01*	1.05	Nevirapine	Toxicity
*HLA-DRB1*09:01*	2.63	Allopurinol	Toxicity
*HLA-DRB1*10:01*	1.05	Nevirapine	Toxicity
*HLA-DRB1*11:01*	5.53	Antiinflammatory agents, Non-steroids	Toxicity
*HLA-DRB1*13:02*	2.89	Allopurinol	Toxicity
*HLA-DRB1*14:01*	2.37	Allopurinol	Toxicity
*HLA-DRB1*15:01*	9.21	Amoxicillin-Clavulanate	Toxicity
		Dapsone	Toxicity
*HLA-DRB1*15:02*	0.26	Allopurinol	Toxicity
*HLA-DRB1*16:01*	0.79	Flupirtine	Toxicity
*HLA-DRB1*16:02*	5.00	Phenytoin	Toxicity
		Dapsone	Toxicity
*HLA-DQB1*02:01*	10.26	Infliximab	Toxicity
*HLA-DQB1*02:02*	9.47	Acetaminophen	Toxicity
		Pegaspargase	Toxicity
*HLA-DQB1*05:01*	7.89	Nevirapine	Toxicity
*HLA-DQB1*05:02*	0.79	Flupirtine	Toxicity
		Allopurinol	Toxicity
*HLA-DQB1*06:01*	0.26	Antithyroid Preparations	Toxicity
*HLA-DQB1*06:02*	9.21	Amoxicillin-Clavulanate	Toxicity
		Influenza vaccines	Toxicity
*HLA-DQA1*01:03*	4.74	Antithyroid Preparations	Toxicity
*HLA-DQA1*02:01*	11.05	Lapatinib	Toxicity
		Azathioprine-Mercaptopurine	Toxicity
